# On the Supposed Function of the Skin as an Absorbent

**Published:** 1827-08

**Authors:** Henry Robertson

**Affiliations:** Author of a Work on the Natural History of the Atmosphere, &c.


*On the supposed Function of the Skin as an Absorbent.* By Henry Robertson, M.D. Author of a Work on the Natural History of the Atmosphere, &c.

The prevention of disease is a part of the physician's duty, next in importance to its successful treatment; but preventive measures can only be founded on the basis of philosophical principles, and an accurate knowledge of the nature of exciting causes, and of their operation. The former I have already fully discussed in my work on the Atmosphere; so far as regards the causes of febrile diseases, I have also adduced what I consider satisfactory proofs that miasmata and contagion act upon the system through the medium of the lungs: my present object is more particularly to show that the skin affords no medium for infection.

The excretory or exhaling power of the skin is fully and satisfactorily treated of by Mr. Cruickshanks: indeed, several of his experiments may be considered as proving that the excretory or exhaling power of the skin is its principal effect on the circulating mass. But this author says not a word of its supposed absorbing powers; and if it can be shown that it does not, in a healthy state possess the power of inhaling or absorbing matters applied to the surface, the notion that certain contagions reach the system through that medium must be abandoned; and so far we shall have got into the right course of ascertaining the channel through which certain volatile poisons infect our bodies.

It has been urged by those who support the opinion that the skin performs an absorbing function, that its lymphatics have been traced opening on the surface: these, of course, could only have been seen on the dead subject, and by the use of quicksilver injection, which, by its weight, might well be supposed to make its way through the unresisting and delicate texture of these vessels in such circumstances, and with an unopposing scarf skin. The orifices of lymphatics opening on the surface have never been detected, either in the living or dead body, by examination with glasses possessing the highest magnifying powers.

The next alledged confirmation of the absorbing function of the skin, is the well-known fact that strangury follows, in many instances, the application of cantharides to any part of the surface; whence it is argued that absorption has taken place by the skin. The fact of absorption in this case, and its effects, will not be disputed; but the manner in which this is accomplished admits, in my opinion, of a more satisfactory explanation; for the strangury never occurs till the cantharides have produced irritation, whereby the scarf-skin is rendered so porous that the absorbents of the cutis are within reach of the plaster, and, being highly excited by inflammation, readily absorb matters that would be rejected in their quiescent state: but whether this is effected by the lymphatics or by minute venous ramifications, it is impossible to say. Another thing to be attended to in this question is, that while the vesicle is entire, no strangury takes place. This only ensues either in peculiar habits before the blister rises, or after the vesication is ruptured. Moreover, every nurse knows that a piece of gause interposed between the blister and the cuticle commonly prevents strangury, though it is no impediment to the ordinary effect of cantharides in inflaming the skin. According to my experience, strangury is more apt to supervene from a blister plaster laid upon the scalp, than when applied to any other part of the skin; and anatomists find fewer absorbents on the scalp than on the surface generally. But this is explained by attending to the shape of the head, where if a plaster is applied, even of the most moderate size, one portion of it necessarily lies flat on the surface, as the serum distends only the most depending portion of the vesication.

The next fact adduced in support of the supposed absorbing function of the skin is taken from the practice of local applications of certain matters to the surface, which are thereby carried into the system;* and chiefly from the effect of mercurial frictions, by which the system becomes imbued with that mineral. In opposition to this opinion it is to be observed, that, except in matters of considerable acrimony, by which the cuticle is affected, the mere application of substances upon the surface is never followed by an absorption of any portion of them; and, in regard to the case of frictions with mercurial ointment, it is very well known that, even in people the most susceptible to the impressions of this mineral, no sensible effect is produced in the system till after considerable friction has been employed, and in an atmosphere of a certain temperature. In other instances, the frictions, together with the acrid nature of the substance, occasion a number of partial inflammations, by which the absorbent vessels of the skin, thus exposed and irritated, may possibly take up a portion of the mineral, and convey it to the circulation. However, from repeated experience on this point, I now am convinced that much delusion exists on the supposed power of the absorbents of the skin in imbibing mercury by friction. It is very well known that quicksilver, either in its pure or compound state, is an extremely volatile substance, even at the ordinary temperature of the air, if it is exposed to agitation, as was experienced in the melancholy occurrence which took place on board the Triumph and Phipps's men of war off Cadiz, in 1810, and in several other familiar instances: accordingly I have, by repeated and varied experiments, found that I never could produce a mercurial affection of the system in any one by friction alone, if the temperature of the air was very low, and access to a fire during the operation prevented, when the common mercurial ointment was employed, unless a certain degree of inflammation in the part rubbed had been produced; and even then the affection of the system was much more tardy in coming on, than when the friction was performed in a warm room, or when some volatile matters, as camphor, or a small portion of any essential oil, was mixed with the ointment. This has been the uniform result, even in men so susceptible of the effect of mercury that two or three frictions by the fire were sufficient to affect the gums. But what I consider places this question in a clearer point of view is, that I have met with two instances in servants who were employed to apply mercurial friction to others, and who were thereby affected with the usual symptoms of that mineral on the system, although their hands, otherwise hard, were carefully protected with

* Our readers will find some remarks on the application of medicines to the skin, in the COLLECTANEA of the present Number.—ED.

oil-skin gloves. I have likewise seen delicate women complain of slightly tender gums and a coppery taste, who had occasion to be much with people under the use of mercury for hepatitis. From these facts, therefore, I am persuaded that the affection of the system supervening upon friction with mercurial medicines is an illusion, on the supposition that it is occasioned by cuticular absorption. On the contrary, I am decidedly of opinion that, in all the above instances, the affection of the system is the consequence of the respiration of mercurial vapour, occasioned by the friction and the heat, whereby the mercury is in a certain degree volatilised : and I consider the two last cases I have adduced as decisive of this opinion.

The favourers of the notion that the skin in a sound state performs an absorbing function adduce, as a proof of their opinion, the well-known fact that thirst is quenched by taking a bath; and this they suppose to be effected by an absorption of the water by the lymphatics of the skin. Upon this fact I shall limit what I have to say to that which takes place in a cold bath; as, in the tepid or hot bath, without precautions are taken to exclude the vapour from having access to the organs of respiration, the experiments must go for nothing, as the absorbing power of the pulmonary system is now determined beyond the possibility of doubt. In speaking of the effect of the cold bath in quenching thirst, the various causes of that sensation must be taken into consideration. Thirst, in a state of health, is occasioned by an increased determination to the skin, either by the temperature of the air or exercise, whereby the thinner portion of the circulating fluid passes off in too great proportion by transpiration, or perhaps more frequently depends on the comparatively indigestible nature of the aliment taken into the stomach. In every instance it is marked by an increased temperature of the skin, and is attended with a corresponding tension in the circulating vessels. The former of these symptoms is probably in consequence of the sympathy between the skin and the digestive organs; therefore the diminution of the heat of the surface by cold bathing acts indirectly upon the alimentary canal, and consequently relieves the feelings of thirst when it arises from either of the causes above mentioned. But it is to be remarked that the same effect is produced whether the bath is of fresh or of salt water, and the latter we know, even admitting its absorption by the skin, is but ill adapted for allaying the sensation of thirst. But, in proof that no absorption takes place by the skin in such cases, I shall refer to the accident that befell

Lieutenant Shannon, related in the Gentleman's Magazine. This gentleman fell into the shaft of an old coalpit, where he remained several days up to the middle in water; yet the greatest misery he endured during all this time was occasioned by the overpowering sensation of thirst he experienced. In addition to the above, I refer to the Aphorisms of SANCTORIUS, in his work on Medical Statics, (section 2d, "De Aere et Aquis.") Indeed, the result of this philosopher's experiments fully bear me out in the explanation I have given of the effects of the cold bath in quenching thirst. I may also bring forward, in proof of the opinion stated, that the skin performs no absorbing function in its healthy condition, the different cases of diabetes on record, where the quantity of urine exceeded considerably the amount of the ingesta, notwithstanding the cuticle was smeared with unctuous matters; and other cases, where the whole surface was covered over with a varnish; as under these circumstances it can never be supposed that the increased quantity of urine was the consequence of absorption by the skin.

There are certain diseases that infect the skin, which are imagined to be the consequence of absorption, as psora and primary syphilis; but this notion seems to be the result of a superficial view of the question; for were these, or any other similar local disease, the effect of absorption, in place of a circumscribed and tardy ulceration, the entire branch of the lymphatics leading from the local affection must have become simultaneously diseased in a similar manner. These diseases most probably originate from the peculiar matter of each exciting a specific irritation and inflammation on that part of the surface to which they are applied, while no absorption takes place till the matter generated is mixed with the fluids of the body. Some diseases, as tinea, are the consequences of a morbid action originating in the skin itself, whereby a matter is generated that acts upon the sound portion of the skin as a corrosive, thus producing ulceration. In this way psora commences on those parts of the body where the cuticle is most delicate; and syphilis primarily attacks those places where, from the extreme tenuity of the cuticle, the skin itself is more exposed to be affected with that or other animal poisons. But in all such cases it appears to me that the cuticle is previously penetrated by these poisons, by which the cutis is thus affected by irritation, independently of absorption.

Indeed, every surgeon meets with daily instances where plaster bandages are loosened and thrown off the surface, in consequence of an aqueous exudation under them; which surely could not happen were the skin, as supposed, possessed of absorbing powers.

I shall conclude these observations by referring to Dr. CURRIE's book on the use of Cold Ablution in Fevers,—to the Dictionnaire des Sciences Medicales, (article Peau,)—and to the second volume of my work on the Atmosphere, for further information on this important question.

Having thus stated some particulars which appear to me of considerable importance in a practical point of view, and which forcibly militate against the supposed absorbing power of the skin, I shall conclude this paper by subjoining two cases which, in my opinion, afford fresh proof of what I have many times advanced, that the volatile poisons find an inlet to the body through the pulmonary system only.

A lady in her sixtieth year, the widow of a person of rank, and the mother of several children, who had through life been very healthy, with the exception of slight nervous symptoms, was, during the winter, taken with a constant micturition, attended with but little irritation, and the secretion was healthy in appearance and quantity. This affection rapidly increased to such a degree that her sleep was frequently broken thereby, and her general health began to be affected. At this time I was consulted. I found that the complaint was always most troublesome during the night; but I could not ascertain any one fact that would lead me to divine its cause, and my prescriptions were continued for some time without giving much relief, till one morning, when I was called to visit my patient much earlier than usual, in consequence of her having passed a night almost deprived of rest, from the urgency of her complaint. I then ascertained, for the first time, that as her bed was placed at some distance from the fire, and the weather being extremely cold, she had always a large pan of burning embers at her bedside while she was undressing; that, on the preceding night, the pan of live embers had at the usual hour been carried to the bedroom, but the lady had remained in the drawing-room rather later than usual, as she felt better in all respects; but she had been but a short time in bed before an aggravation of the leading symptom supervened. Having this explanation of the circumstances, I was convinced that the inhalation of the gas arising from the burning embers was the cause of my patient's illness, and having pointed out to her the pernicious consequences that arise from warming apartments by such means, the practice above mentioned was discontinued, and my patient, in the short space of forty-eight hours, felt no other inconvenience than debility from a month's illness.

Shortly after the occurrence of the above, a young person, attendant of a lady of my acquaintance, consulted me on account of a burning of her face, that generally came on towards evening, with frequent micturition. Having the experience of the former case, I found by my questions that she was in the practice of warming her mistress's bed with a pan of burning wood-ashes, and, as she slept in a room adjoining, she always carried the pan there afterwards, and left it in order to warm her own apartment. I then directed her to discontinue this practice; and, by a little attention to diet, with some aperient medicines, her complaint very shortly disappeared.

I state these cases from the idea that the excitement of the urinary organs in both could only have arisen from the absorption of the carbonic acid gas by the lungs; and that, by analogy, we may conclude that these organs must have a similar power over other gaseous matters, such as contagions, marsh effluvia, &c. that may happen to be mixed in the atmosphere, and which are much too subtile to have an effect on the system through the skin, could we even admit that it enjoys the power of absorption, or that our apparel offers no obstacle to the successive access of such matters to the surface of our bodies.

Philosophers of late years have been most laudably occupied in attempts to unravel the functions of the lungs, from which our knowledge of the animal economy has been importantly augmented. But at the same time it is to be lamented that great uncertainty prevails regarding the particular structure of these organs, and, until this is fully ascertained, our acquaintance with the functions must be limited in a proportionate degree. One daily hears the air-cells spoken of in the human lungs, as if they could be as distinctly demonstrated as in those of the tortoise, and although the most indefatigable anatomists deny their existence.

The sympathy that exists between the skin and every other organ of the body, admits of daily illustration in the practice of every physician; and, although I am convinced that the skin in a healthy condition does not possess absorbent powers, nevertheless, as an excretory organ, it often supplies by that office a similar function to that of the lungs, when any thing interferes with their due action. I have shown, in my observations on respiration, in my work on the Atmosphere, that the functions of the lungs are materially affected during digestion; for instance, that the air expired after a full meal is more charged with carbonic acid than at other times. Sanctorius, in Aphorism xi. de Cibo et Potu, has corresponding observations respecting the skin.

From these facts some inferences of utility may be drawn, by those whose duty carries them into situations abounding with contagion, or where other gaseous matters inimical to health are mixed with the atmosphere. The most effectual mode of guarding against the consequences attending the inspiration of such matters, is to defend the mouth and nostrils;* or, perhaps, a close-wove gause veil, so ample as, when thrown over the head, to cover the shoulders and breast, might be usefully adopted as a substitute for the above.

Although I have endeavoured, by the foregoing facts, to shew that the skin does not possess the power of absorbing matters applied to it while in its sound and healthy condition, I do not thereby mean to deny the effect of certain remedies applied to that organ, by an impression on its sensitive or nervous power, as in the case of narcotics, fomentations, refrigerants, &c.; for these and all similar matters operate directly upon the nerves of the skin, and do not produce their effects through absorbent organs.

---

[In several of our recent Numbers, we have given papers from the American Journals. Some of these periodicals contain numerous original communications of interest, and it is our intention hereafter to insert such of them as are of sufficient importance and as our limits will admit of, immediately after the papers of our own contributors.]

* See Medical Repository, December 1818.

